# PAK6 increase chemoresistance and is a prognostic marker for stage II and III colon cancer patients undergoing 5-FU based chemotherapy

**DOI:** 10.18632/oncotarget.2803

**Published:** 2014-11-07

**Authors:** Jian Chen, Huijun Lu, Dongwang Yan, Feifei Cui, Xiaoliang Wang, Fudong Yu, Yingming Xue, Xiaodong Feng, Jingtao Wang, Xiao Wang, Tao Jiang, Meng Zhang, Senlin Zhao, Yang Yu, Huamei Tang, Zhihai Peng

**Affiliations:** ^1^ Department of General Surgery, Shanghai First People's Hospital, School of Medicine, Shanghai Jiao Tong University, Shanghai, People's Republic of China; ^2^ Department of Pathology, Shanghai First People's Hospital, School of Medicine, Shanghai Jiao Tong University, Shanghai, People's Republic of China; ^3^ Basic Medical College, Taishan Medical University, Tai'an, People's Republic of China; ^4^ Department of Anal-Colorectal Surgery, General Hospital of Ningxia Medical University, Yinchuan, People‘s Republic of China

**Keywords:** p21-activated kinase 6, Colon cancer, 5-fluorouracil, Chemoresistance

## Abstract

p21-Activated kinase 6 (PAK6) has been implicated in radiotherapy and docetaxel resistance. We have further evaluated PAK6 as a predictor of 5-fluorouracil (5-FU) treatment response in colon cancer. Here we report that in colon cancer PAK6 promotes tumor progression and chemoresistance both *in vitro* and *in vivo*. In the clinical analysis, PAK6 was overexpressed in 104 of 147 (70.75%) stage II and III patients who received 5-FU based chemotherapy after surgery. Multivariate Cox regression analysis indicated that PAK6 was an independent prognostic factor for overall survival (*P* < 0.001) and disease-free survival (*P* < 0.001). Colon cancer cell lines showed increased PAK6 expression upon 5-FU treatment. In PAK6-knockdown cells treated with 5-FU, cell viability and phosphorylation of BAD decreased, and the number of apoptotic cells, levels of cleaved caspase 3 and PARP increased compared to control cells. The opposite was observed in PAK6 overexpressing cells. Short hairpin RNA knockdown of PAK6 blocked cells in G2-M phase. Furthermore, Animal experiments results in vivo are consistent with outcomes in vitro. This study demonstrates that PAK6 is an independent prognostic factor for adjuvant 5-FU-based chemotherapy in patients with stage II and stage III colon cancer.

## INTRODUCTION

Colon cancer is the third leading cause of cancer deaths worldwide [1]. Although surgical resection alone is potentially curative, local or distant recurrences develop in many patients. 5-fluorouracil (5-FU)-based systemic adjuvant chemotherapy has been suggested for patients with a high risk of recurrence, and has been shown to be beneficial in a number of trials [2]. Despite these advances, the role of chemotherapy in colon cancer is still debated, especially for stage II and III colon cancer patients [3-5]. Recently, the development of 5-FU resistance during the course of treatment, which is an important cause of colon cancer therapy failure, has become more common [6-8]. However, laboratory studies investigating the molecular biology of cancer initiation and progression have so far provided little information regarding the molecular mechanisms underlying chemotherapy resistance. In order to more effectively target colon cancer treatment in the future, it is important to identify patients who are at risk of recurrence while receiving 5-FU-based therapy. As such, exploring useful biomarkers for 5-FU-based chemotherapeutic sensitivity in patients with colon cancer is clinically important.

p21-Activated kinases (PAKs) are a family of Rac/Cdc42-associated Ser/Thr protein kinases that phosphorylate small GTPase effectors. They have been implicated in the regulation of multiple cellular functions, including actin reorganization, cell motility, gene transcription, cell transformation, apoptotic signaling, and more recently, radiotherapy and chemotherapy resistance signaling [9-12]. PAK6 is the most recently identified, and least well understood, member of the PAK family of proteins. In recent years, PAK6 has been shown to be principally overexpressed in the brain and testes, although low levels have been detected in numerous tissues, including the prostate and breast [13]. Knockdown of PAK6 expression results in the inhibition of prostate cancer growth and enhanced chemosensitivity to docetaxel [14]. When combined with irradiation, inhibition of PAK6 results in significantly decreased prostate cancer cell survival [9]. However, in colon cancer patients who receive adjuvant chemotherapy after tumor resection, the role of PAK6 remains unclear. The aim of this study was to evaluate whether PAK6 can influence 5-FU-based chemotherapeutic sensitivity in colon cancer cells, and determine whether PAK6 expression is a useful marker for guiding 5-FU adjuvant therapy after curative resection of colon cancer. In this study, we used quantitative real-time polymerase chain reaction (qPCR), western blotting, and immunohistochemistry (IHC) to assess PAK6 expression, and found that it was markedly increased in colon cancer tissues relative to normal colon epithelium. We subsequently sought to assess the effects of PAK6 expression on the 5-FU-based chemotherapy in colon cells by downregulating and overexpressing *PAK6* mRNA and protein, and tried to elucidate the possible mechanisms underlying PAK6 mediated chemotherapy resistance.

## RESULTS

### Aberrant overexpression of PAK6 in colon cancer tissues

**Figure 1:**
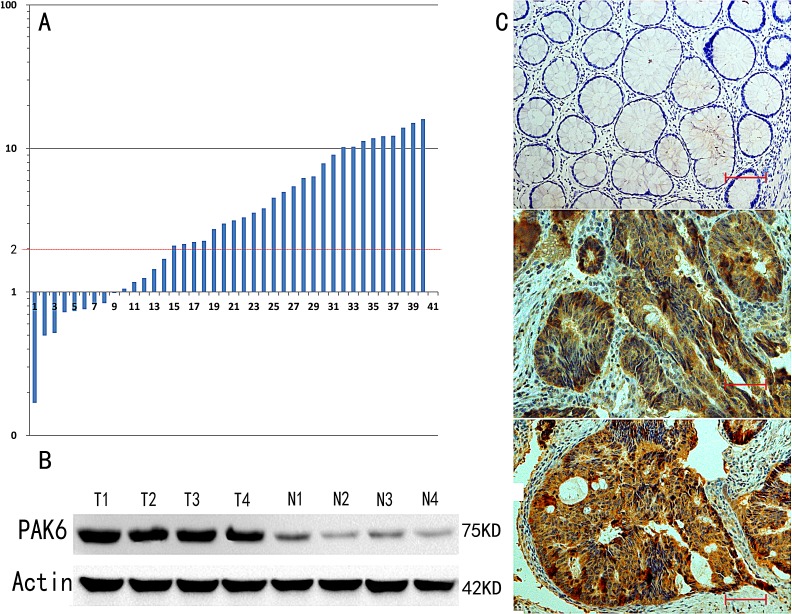
p21-Activated kinase 6 (PAK6) expression is increased in colon tumors A: At least a 2-fold increase in *PAK6* mRNA levels compared to noncancerous tissue was observed in 26 (65%) of 40 colon cancers. The logarithmic scale 2^−ΔΔCt^ was used to represent the fold change in the quantitative real-time polymerase chain reaction (qPCR) analysis. B: Western blotting confirmed that higher PAK6 protein expression was observed in colon cancer samples compared to the matched adjacent non-tumor tissues. C: Immunohistochemical staining for PAK6 expression in normal and colon cancer tissues (200 ×, Bar = 100 um).

Of the 40 randomly selected paired cases that were used to evaluate *PAK6* mRNA and protein expression, 26 (65%) colon cancers had at least a 2-fold increase in *PAK6* mRNA levels compared to the adjacent noncancerous tissues. This difference in *PAK6* mRNA expression was significant (*P* < 0.001, Fig. 1A). Subsequent western blotting confirmed that the level of PAK6 protein was also higher in the colon cancer samples than in the matched adjacent non-tumor tissues (Fig. 1B). These results suggest that PAK6 is upregulated in colon cancer.

### Association between PAK6 expression and the clinical features of colon cancer

To determine whether PAK6 expression correlated with colon cancer progression, the expression of PAK6 was determined by IHC in 147 stage II and III patients who accepted 5-FU-based chemotherapy after radical colectomy. As shown in Fig. 1C, PAK6 protein was localized in the cytoplasm of cancer cells, with strong staining in the cancer mucosa. We found that PAK6 was dramatically upregulated in 104 of 147 (70.75%) primary colon cancer patients specimens, but was either absent or only minimally expressed 9 of 147 (6.12%) in the adjacent normal colonic tissue. The IHC staining quantitative analysis is summarized in Table 1. We observed that the level of PAK6 expression was closely correlated with tumor differentiation (*P* = 0.002) and recurrence (*P* < 0.001) in colon cancer patients. Collectively, these data indicate that PAK6 may be involved in colon cancer progression.

**Table 1:** PAK6 immunohistochemical staining for protein expression in colon cancer

		PAK6 expression			
variable	n=147	Negative (n=43)	weak (n=69)	Positive (n=35)	P value
Age					0.929
<65	61	19	28	14	
≥65	86	24	41	21	
Gender					0.169
Male	66	20	26	20	
Female	81	23	43	15	
Location					0.976
Right	79	23	38	18	
left	68	20	31	17	
T stage					0.913
T1+ T2	6	2	3	1	
T3+ T4	141	41	66	34	
N stage					0.608
N0	69	20	35	14	
N1+N2	78	23	34	21	
AJCC stage					0.652
II	67	19	34	14	
III	80	24	35	21	
Differentiation					0.002*
high	68	29	29	10	
low	79	14	40	25	
Vessel invasion					0.720
𠀃No	136	41	63	32	
𠀃Yes	11	2	6	3	
Tumor recurrence					<0.001*
No	85	37	37	11	
Yes	62	6	32	24	

* *P* < 0.05 indicates a significant association among the variables.

### High PAK6 expression is associated with a poor clinical outcome in human colon cancer

**Figure 2:**
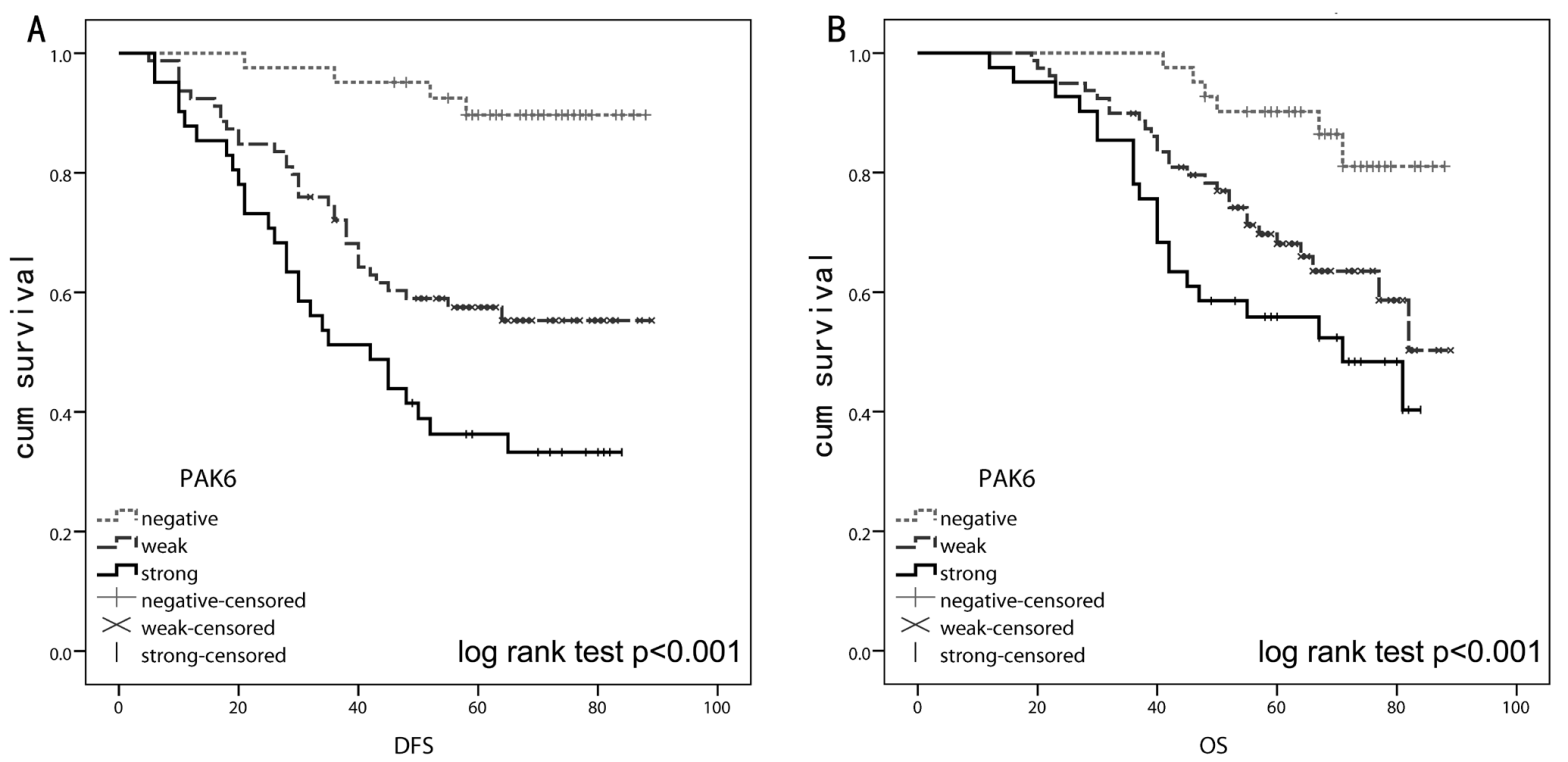
Kaplan-Meier and log rank test analysis of survival for 147 stage II and III colon cancer patients who received 5-fluorouracil (5-FU)-based chemotherapy after surgery A: Lower disease-free survival (DFS) was observed in patients with higher PAK6 expression (*P* < 0.001). B: Overall survival (OS) was significantly better in patients with PAK6-negative tumors than in patients with PAK6-positive tumors (*P* < 0.001).

Results from the IHC analysis of 147 stage II and III tissues indicated that there was a significant difference between the PAK6-positive and PAK6-negative groups in the number of patients who developed primary colon cancer recurrence after 5-FU-based chemotherapy; more of the patients with PAK6-positive tumors underwent subsequent recurrence than the patients with PAK6-negative tumors (*P* < 0.001). Positive PAK6 expression (weak and strong) was associated with a 2.221-fold increased risk of recurrence (hazard ratio [HR], 2.221; 95% confidence interval [CI] 1.532–3.220]; *P* < 0.001). We used the Kaplan-Meier analysis to show that the expression of PAK6 was significantly correlated with the disease-free survival (DFS) of colon cancer patients (log-rank test, *P* < 0.001; Fig. 2A). Patients with PAK6-positive tumors had significantly lower 5-year overall survival (OS) than those with PAK6-negative tumors (HR, 2.335; 95% CI, 1.203–4.535; *P* < 0.001; Fig. 2B). In multivariate analysis using the clinicopathological variables such as differentiation grade and vessel invasion, the expression of PAK6 was an independent prognostic marker to predict patient outcomes (Table 2); this suggests that PAK6 is of clinical significance in the diagnosis and prognosis of patients with stage II and III colon carcinomas who are treated with 5-FU-based chemotherapy.

**Table 2:** multivariate Cox proportional hazards models for disease-free survival (DFS) and overall survival (OS)

	Multivariate analysis (DFS)		Multivariate analysis (OS)	
	P value	HR (95%CI)	P value	HR (95%CI)
Gender(female vs. Male)	0.707	1.107(0.652, 1.880)	0.658	1.142(0.636, 2.050)
Age(＞65 years vs.≤65 years)	0.470	1.211(0.720, 2.035)	0.287	1.372(0.766, 2.456)
Tumor stage (III vs. II)	0.242	3.412(0.437,26.636)	0.344	2.804(0.331, 23.765)
Differentiation (Low vs. High)	0.036*	1.841 (1.041, 3.257)	0.012*	2.335(1.203, 4.535)
Tumor location (Left vs. Right)	0.257	1.354(0.801, 2.289)	0.121	1.360(0.761, 2.433)
Vessel invasion (Yes vs. No)	0.106	1.879(0.874, 4.040)	0.043*	2.164(1.025, 4.569)
PAK6 (positive vs. negative)	<0.001*	2.221(1.532,3.220)	<0.001*	2.335(1.203, 4.535)

HR: Hazard ratio; CI: Confidence interval.

* *P* < 0.05 indicated that 95% CI of HR was not including 1.

### 5-FU induces upregulation of PAK6 expression

**Figure 3:**
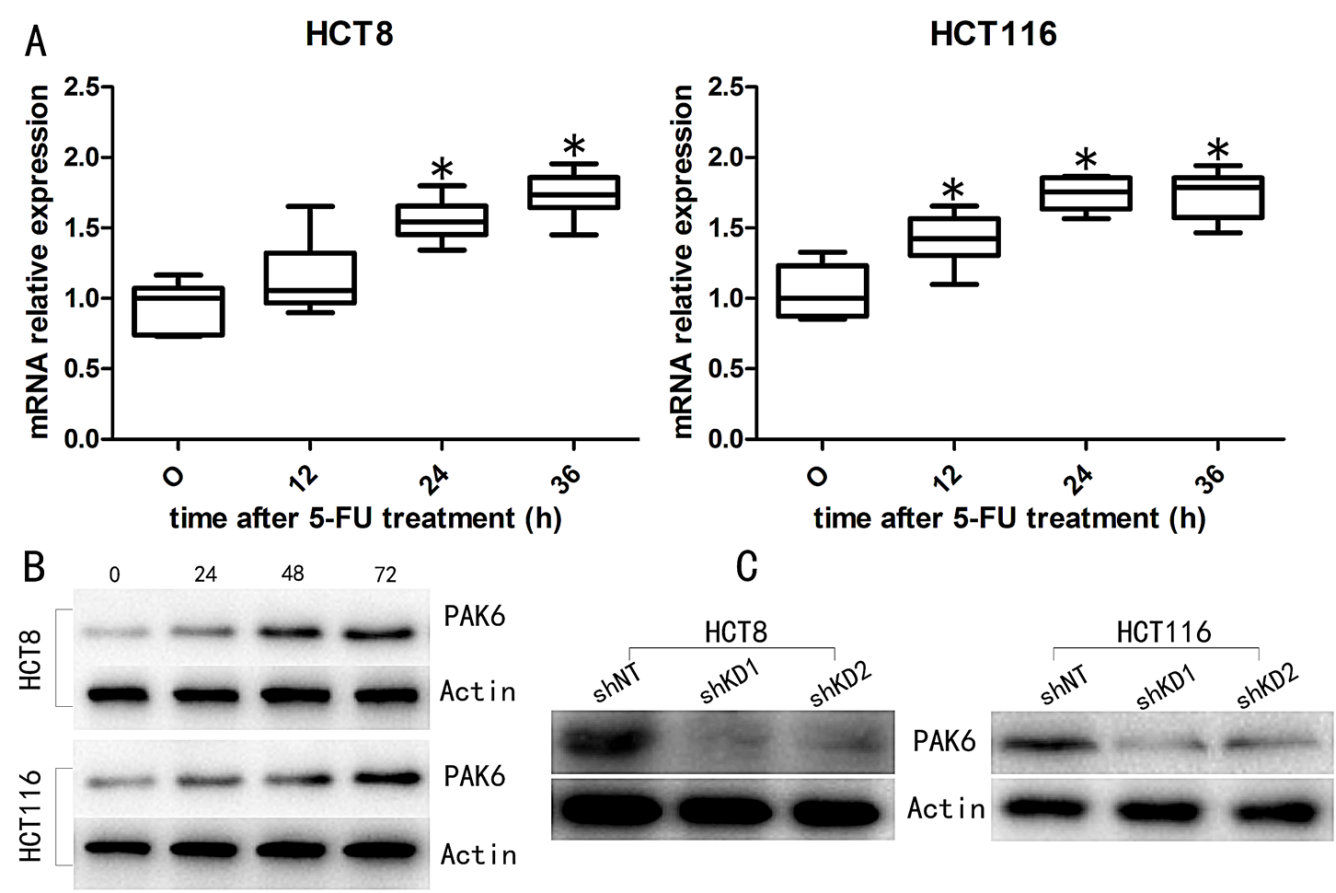
5-FU treatment induces PAK6 expression A: qPCR analysis indicated that *PAK6* mRNA was increased in both HCT8 and HCT116 cells treated with 10 μM of 5-FU for 0, 12, 24, and 36 h. B: The level of PAK6 protein was also increased in HCT8 and HCT116 cells following 5-FU treatment. C: Western blotting confirmed PAK6 knockdown (KD) in HCT8 and HCT116 cell lines. Values are means ± the standard error of the mean (SEM; * *P* < 0.05).

To investigate the role of PAK6 in mediating sensitivity to colon cancer chemotherapy, we used 2 human colon cancer cell lines, HCT8 and HCT116. Western blot analysis showed that both untreated cell lines expressed PAK6 protein. Following treatment with 10 μM 5-FU, a gradual increase in the *PAK6* mRNA levels was observed in both HCT8 and HCT116 cells (Fig. 3A). To examine whether PAK6 protein levels were also altered after 5-FU treatment, western blot analysis was performed. As shown in Fig. 3B, PAK6 levels increased following 5-FU treatment in both cell lines, which was similar to our qPCR data. SW480 cells, which are less malignant than HCT116 and HCT8 cells, showed very weak PAK6 expression before and after 5-FU treatment (data not shown).

### PAK6 increases cell viability

**Figure 4:**
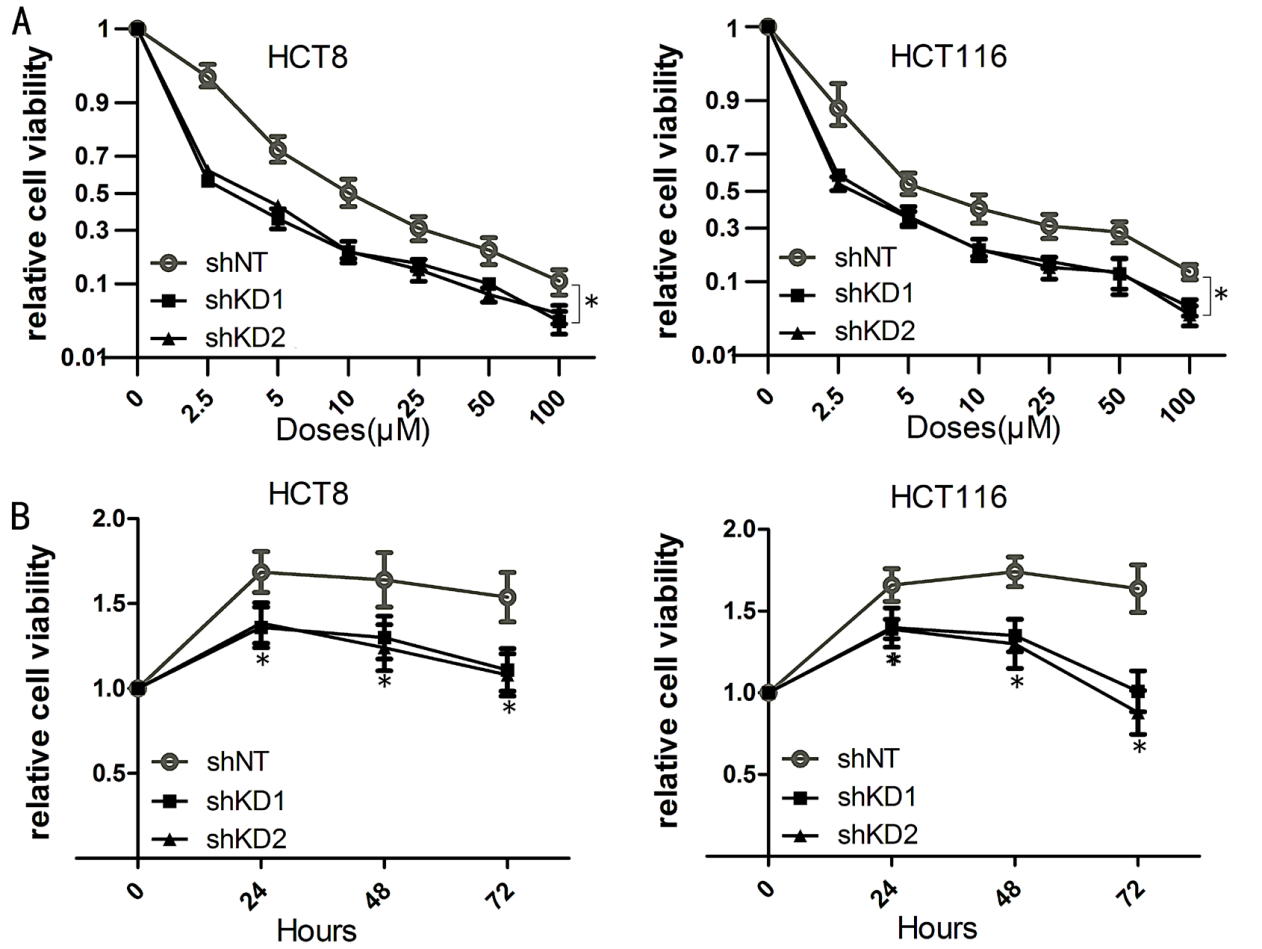
Inhibition of PAK6 decreases cell viability A: Cell viability was reduced by 5-FU treatment in a dose-dependent manner. Cells were treated with 7 concentrations of 5-FU (0 μM, 2.5 μM, 5 μM, 10 μM, 25 μM, 50 μM, and 100 μM) for 48 h. Transduction with PAK6 short hairpin (sh) RNA shifted the survival curves downward for both HCT8 and HCT116 cells. The 5-FU half maximal inhibitory concentrations (IC50s), calculated from 2 independent experiments performed in quadruplicate (n = 8), ± SEM were HCT8 non-targeting shRNA (shNT) IC50 = 12.51 ± 0.05 μM, HCT8 shKD1 IC50 = 2.98 ± 0.05 μM, HCT8 shKD2 IC50 = 3.79 ± 0.04 μM, HCT116 shNT IC50 = 9.22 ± 0.11 μM, HCT116 shKD1 IC50 = 3.09 ± 0.07 μM, HCT116 shKD2 IC50 = 2.67 ± 0.06 μM. B: PAK6-downregulated cell lines showed a significant reduction in cell viability at all 4 time points (0, 24, 48, and 72 h) following 10 μM 5-FU treatment (* *P* < 0.05).

Western blot analysis was used to confirm the efficiency of PAK6 knockdown (KD) and overexpression (Fig. 3C and Fig. S1A). MTT cell viability assays were performed following 5-FU treatment and, as shown in Fig. 4A and Fig. S1B, cell viability was reduced by 5-FU in a dose-dependent manner. PAK6-KD with short hairpin (sh) RNA shifted the survival curves for HCT8 and HCT116 cells downwards; conversely, PAK6 overexpression in SW840 cells increased survival. To determine how PAK6 inhibition affects cell viability, MTT assays were performed on HCT8 and HCT116 cells incubated with 10 μM 5-FU for 0, 24, 48, and 72 h. PAK6-KD cell lines showed a significant reduction in cell viability with all 3 incubation times (24, 48, and 72 h) compared to control cells (Fig. 4B). In summary, targeted inhibition of PAK6 sensitized HCT8 and HCT116 cells to chemotherapy while overexpression of PAK6 increased SW480 chemoresistance. These data indicated that PAK6 mediates colon cancer cell chemoresistance.

### PAK6-KD sensitizes cells to apoptosis

**Figure 5:**
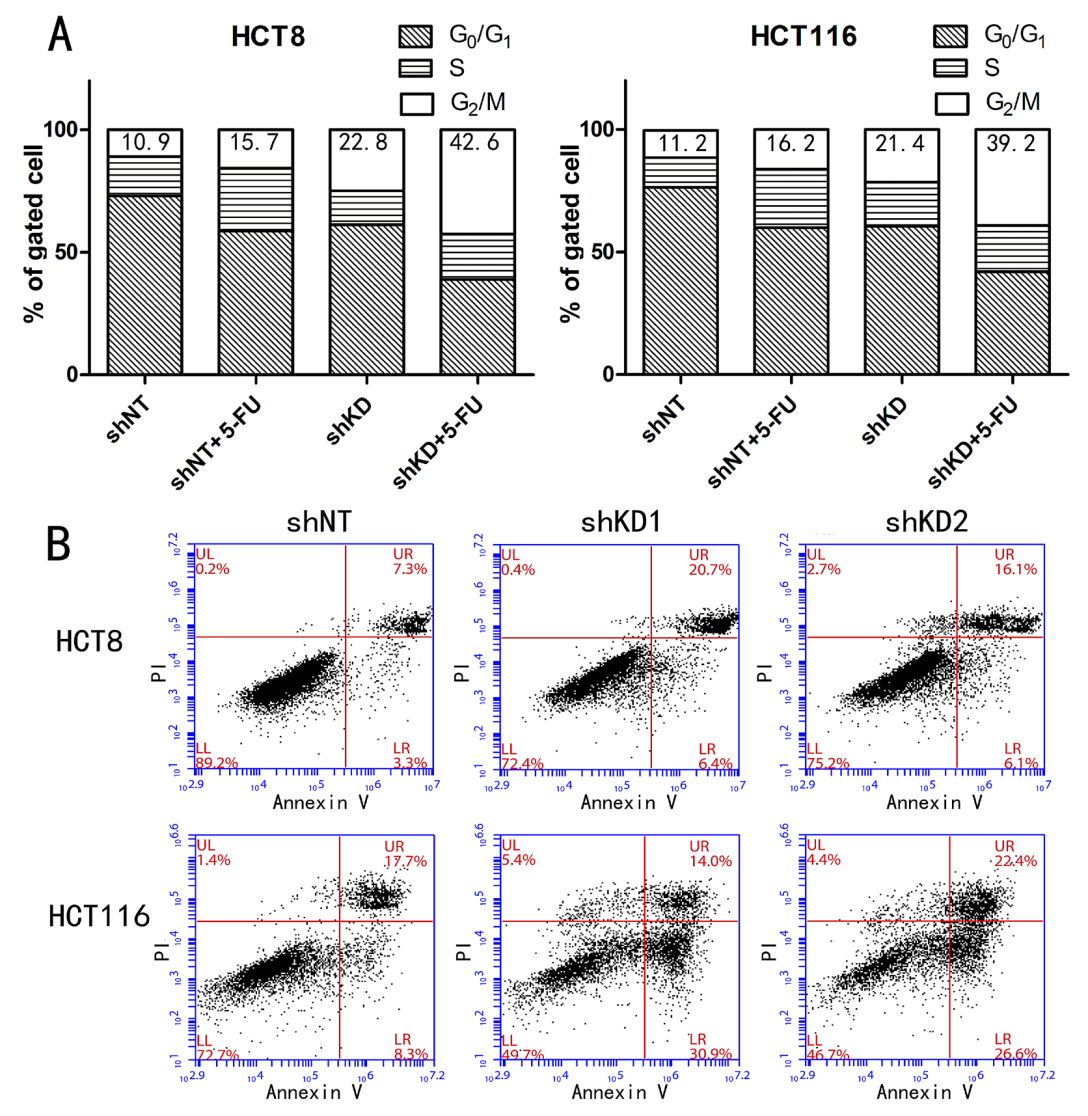
PAK6-KD cells exhibit a greater degree of cell cycle arrest and apoptosis A: PAK6-KD in HCT8 and HCT116 cells resulted in a significant increase in G2/M phase cell cycle arrest. The experiments were performed 3 times, but the data are from a single representative experiment. B: Apoptosis in HCT8 and HCT116 cells treated with 10 μM 5-FU for 48 h was clearly increased in cells in which PAK6 had been downregulated compared to control cells.

We proceeded to explore the role of PAK6 in chemotherapeutic resistance. In both HCT8 and HCT116 cells, transduction with PAK6-shRNA resulted in a significant increase in G2/M-phase cells, and a reciprocal decrease in G0/G1-phase cells, compared to cells that had been transduced with a non-targeting shRNA (shNT; Fig. 5A). This effect was augmented by the addition of 5-FU. However, 5-FU treatment of the shNT control cells increased the proportion of S-phase cells. These data indicate that at the time of 5-FU treatment, the cells were blocked in a more chemosensitive stage of the cell cycle (Table S1).

### PAK6 rescues cells from 5-FU induced apoptosis

**Figure 6:**
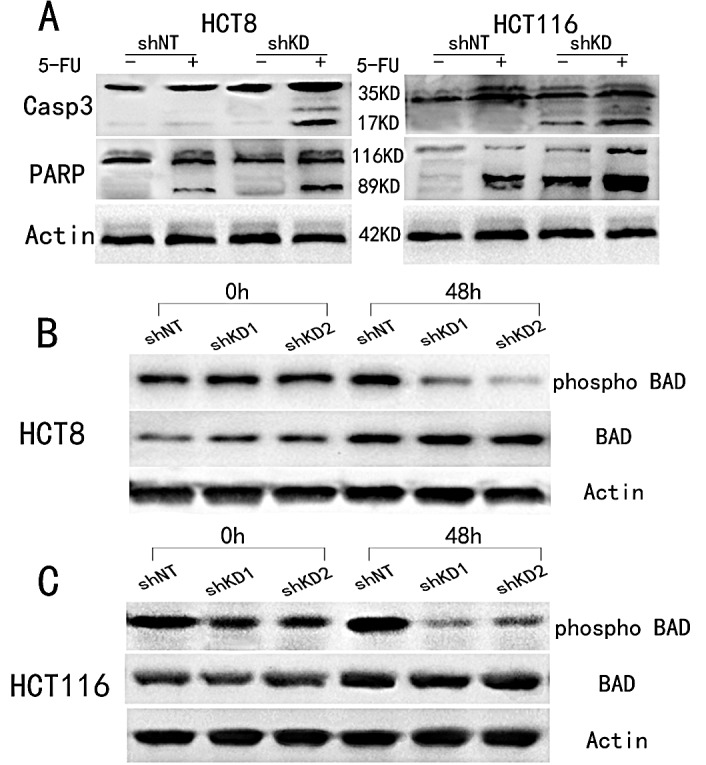
Change in apoptosis-related molecules after treatment with 10 μM 5-FU for 48 h A: Western blot analysis showed that caspase 3 and PARP were cleaved in PAK6-KD cell lines. B: Phosphorylated BAD (p-BAD) was decreased in PAK6-KD HCT8 cells. C: Phosphorylated BAD (p-BAD) was decreased in PAK6-KD HCT116 cells.

Cell survival was assessed using annexin V and propidium iodide (PI) staining after a 48 h incubation with 10 μM 5-FU. In PAK6-KD HCT8 and HCT116 cells, 5-FU treatment induced a 2-fold increase in apoptosis compared to shNT control cells (Fig. 5B); conversely, in SW480/PAK6 cells, PAK6 overexpression lead to a reduction in apoptosis compared with SW480/vector cells. (Fig. S1C). Together, these data indicate that PAK6 overexpression in tumor cells is responsible for resistance to 5-FU-induced apoptosis. To further explore the molecular mechanisms underlying the anti-apoptotic role of PAK6, we examined 2 biochemical markers of apoptosis, poly adenosine diphosphate ribose polymerase (PARP) and cleaved caspase 3, in the shRNA stable cell lines. After a 48 h treatment with 10 μM 5-FU, cleaved PARP and caspase 3 were detected in the PAK6-KD cell lines (Fig. 6A); conversely, in the shNT cell lines, which are resistant to apoptosis, the majority of the PARP and caspase 3 proteins were uncleaved and remained full-length. Similarly, the SW480/vector cells showed higher levels of cleaved PARP and caspase 3 compared to the SW480/PAK6 cells (Fig. S2A). These results suggest that PAK6-KD may confer sensitivity to 5-FU-induced apoptosis. The B-cell lymphoma (Bcl)-2 family of proteins are known to be closely associated with apoptosis. It has been reported that when Bcl-2-associated death promoter (BAD) protein is phosphorylated, it ceases to interact with Bcl-2 or Bcl-xL, which prevents the release of cytochrome c and suppresses apoptosis [15]. As shown in Fig. 6B, a significant decrease in phosphorylated-BAD (p-BAD) was observed in PAK6-KD cells treated with 5-FU for 48 h; in control cells a slight increase in p-BAD was observed after 5-FU treatment, and similar results were obtained in HCT116 cells (Fig. 6C). SW480/PAK6 cells showed increased p-BAD compared to SW480/vector cells (Fig. S2B). Taken together, these findings indicate that PAK6 may protect colon cancer cells from 5-FU induced apoptosis.

### PAK6 promotes colon cancer growth *in vivo*

**Figure 7:**
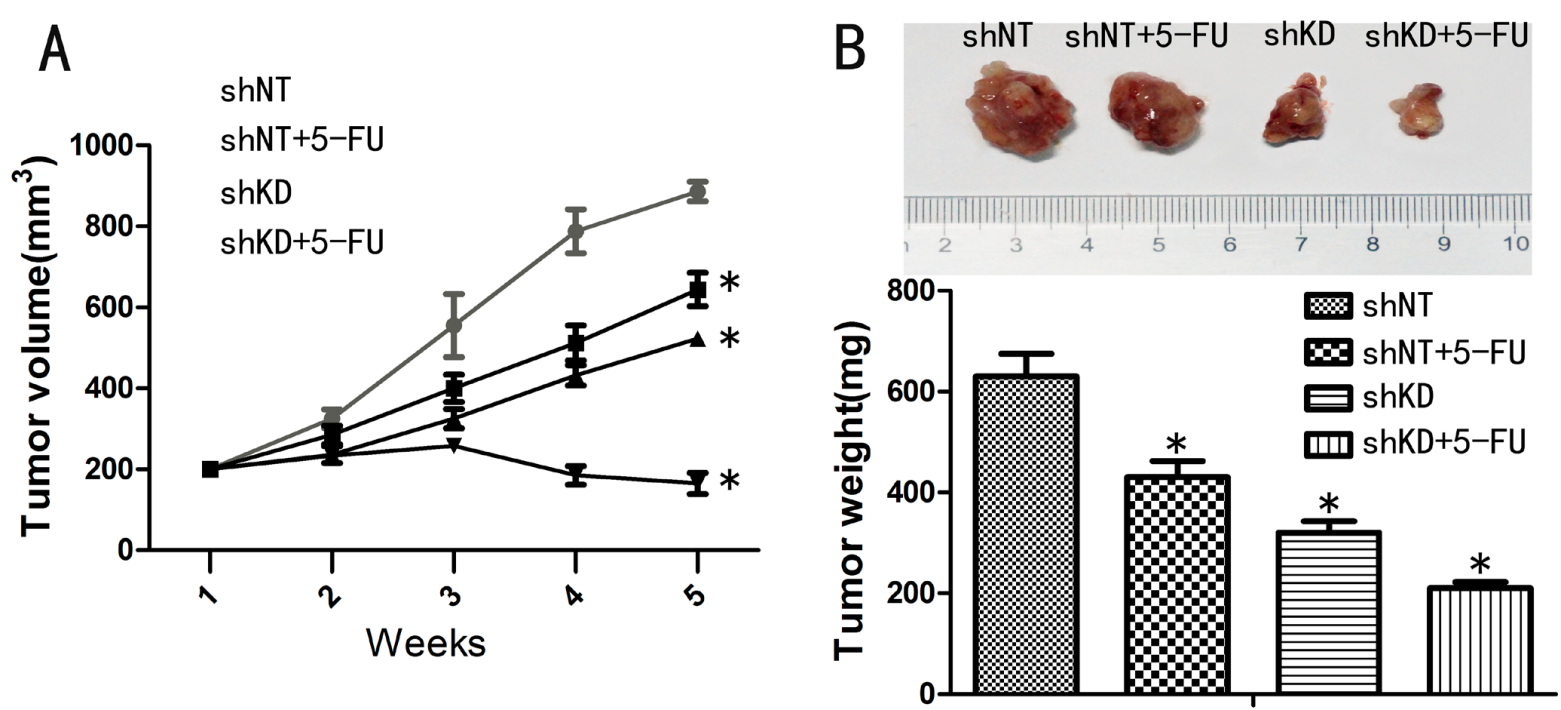
Effect of PAK6-KD and 5-FU on tumor xenograft growth A: Tumor volume curves of mice in different treatment groups. B: Average weight of tumors harvested in the 5th week. Values are the mean ±SEM from HCT8 cells (the results using HCT116 were as the same;* P < 0.05 compared to shNT cells).

Based on the *in vitro* findings described above, we proceeded to investigate the tumorigenicity of PAK6-KD and shNT cells when subcutaneously injected into the abdomen of nude mice that were/were not treated with 5-FU. All of the mice survived the transplantation and appeared healthy. The tumor growth curves and harvested tumor weights are shown in Fig. 7A and Fig. 7B. As anticipated, the tumor growth and tumor weights were significant decreased in the PAK6-KD group compared to the control group. An even more significant reduction was observed in the PAK6-KD group treated with 5-FU. These *in vivo* data are consistent with the *in vitro* results, and confirm that PAK6 overexpression promotes colon cancer progress and 5-FU chemoresistance.

## DISCUSSION

This is the first report showing evidence for PAK6 playing a critical role in colon cancer pathogenesis and chemoresistance. The data presented support the hypothesis that PAK6 could serve as a prognostic indicator of colon cancer outcome in patients with stage II and III disease treated with 5-FU-based adjuvant chemotherapy following curative surgery. Furthermore, the presented mechanistic evidence strongly suggests that dysregulated PAK6 expression confers 5-FU chemoresistance in colon cancer cells. Collectively, our novel clinical and mechanistic data demonstrate that increased PAK6 expression confers a more aggressive phenotype and resistance to 5-FU-based chemotherapy in colon cancer.

Upregulation of PAK6 has previously been verified in primary and metastatic pancreatic cancer, and has been shown to be further increased in tumors that relapse after androgen deprivation therapy [16, 17]. In addition, PAK6 expression has been found to be decreased in renal cell carcinoma, but increased in hepatocellular carcinoma; PAK6 expression is also thought to be a potentially useful marker for the differentiation of human uterine cervical adenocarcinoma from squamous cell carcinoma [18-20]. In the present study, we initially measured PAK6 expression in fresh frozen colon cancer specimens and found that *PAK6* mRNA and protein levels were higher in colon tumor tissues than in the surrounding noncancerous mucosa. These data indicated that PAK6 was upregulated at both the transcriptional and post-transcriptional level. Further validation by IHC showed that PAK6 protein was localized to the cytoplasm of cancer cells, and 104 of 147 (70.75%) primary colon cancers showed positive staining; only 9 of 147 (6.12%) normal colonic epithelium were immunoreactive for PAK6. Thus, we have provided the first clinical evidence that PAK6 might play an important role in the progression of colon carcinogenesis.

Until now, no valid prognostic biomarkers for colon cancer chemotherapy have been established, although several potential molecular predictors of recurrence risk and chemotherapy benefit have been investigated (e.g., KRAS expression, levels of DNA mismatch repair, and p53 expression) [21, 22]. However, so far, only KRAS mutation analysis has been used clinically as a predictive marker for the efficacy of anti-epidermal growth factor receptor antibodies in metastatic disease [23]. The analysis of other known critical colon cancer genes is neither recommended for screening nor used as a prognostic indicator, in particular for stage II and III patients [4, 5, 24]. Patients with stage II colon cancer are considered for adjuvant chemotherapeutic treatment if, based on clinical and pathological evaluation, they are deemed to be at a high risk of relapse [25]. According to the recommendations of the American Society of Clinical Oncology, adverse factors include an advanced T stage, few examined lymph nodes, low tumor differentiation, and tumor perforation [26]. However, these clinicopathological risk factors do not clearly distinguish between patients who have a high or low risk of disease recurrence, and do not predict which patients are likely to specifically benefit from chemotherapy [27]. In the present study, stage II and III patients with high levels of tumor PAK6 expression showed an increased risk of tumor recurrence when treated with 5-FU-based chemotherapy after radical surgery. Furthermore, for stage II and III colon cancer patients, high PAK6 expression was an independent prognostic factor for OS (*P* < 0.001) and DFS (*P* < 0.001). All of these results indicate that PAK6 has the potential to predict 5-FU-susceptibility in patients with stage II and stage III colon cancer. This information could help to inform clinical decisions and target therapeutics to patient subgroups with an increased likelihood of disease recurrence. However, our current study is limited in that it was conducted retrospectively and has limited generalizability because all of the patients were Chinese. As such, further investigation into the potential clinical application of our results to colon cancer therapy should be conducted, especially in regards to improving chemotherapeutic outcomes for stage II and III colon cancers.

5-FU is a commonly used chemotherapeutic agent that damages DNA and inhibits cancer growth by initiating apoptosis [6]. Anti-cancer drug resistance can result from various causes, including alterations in drug influx and efflux, enhanced drug inactivation, and mutation of the drug target [28]. High levels of thymidylate synthase expression [29], increased deoxyuridine triphosphatase activity [30], and MLH1 gene methylation [31] have all been reported to lead to 5-FU drug resistance; as such, multiple factors appear to be able to contribute to 5-FU resistance [32], and it is likely that many mechanisms of 5-FU drug resistance remain to be demonstrated. An effective method to identify potential therapeutic targets associated with chemoresistance is to compare gene expression in human colon cells before and after chemotherapy [33]. The present study has shown that PAK6 is significantly upregulated in both HCT8 and HCT116 cells following 5-FU treatment. Therefore, PAK6 overexpression may influence the response of colon cells to 5-FU chemotherapy. To clarify the functional role of PAK6 in chemotherapy, we used an shRNA-based strategy to stably downregulate PAK6 in colon cancer cells that express high levels of the protein, and overexpressed PAK6 in colon cancer cells that express low levels of the protein. We showed that attenuation of PAK6 protein by shRNA decreased cell viability, and overexpression of PAK6 increased viability. Moreover, transduction of colon cancer cells with PAK6 shRNA resulted in cellular arrest in G2-M stage, a more chemosensitive stage of the cell cycle. Therefore, at the time of 5-FU treatment, cells were arrested in a more chemosensitive stage of the cell cycle.

A key mechanism through which cancer cells become resistant to chemotherapy is by the disruption of the pathways that lead to apoptosis [34]. In this study, it was shown that in colon cancer cells, PAK6 inhibition significantly increased the extent of 5-FU induced apoptosis and increasing PAK6 protein expression decreased apoptosis, supporting the notion that PAK6 plays a role in apoptosis evasion. To further explore the mechanism by which PAK6 mediates chemoresistance, we examined PARP and caspase 3 cleavage and BAD phosphorylation. In PAK6-KD cells treated with 5-FU, significantly more cleaved PARP and caspase 3 and less p-BAD were detected compared to control cells; in PAK6 overexpressing cells, the opposite was observed, indicating that PAK6 may confer chemoresistance through anti-apoptotic pathways.

In summary, our results indicate that a combination of PAK6 inhibition and 5-FU treatment results in significantly decreased survival in colon cancer cells, which can be restored by PAK6 expression. These findings highlight the potential role that PAK6 may play in chemoresistance and indicate that PAK6 could be an effective target for 5-FU sensitization in colon cancer, although further research is required to confirm our findings. Finally, we were able to confirm the role of PAK6 in colon cancer progression in animal models. Our study indicated that tumor growth and tumor weights were significantly decreased in PAK6-KD tumor xenografts, especially when treated with 5-FU. These results were consistent with our *in vitro* results and suggest that PAK6 is a critical protein responsible for 5-FU-based chemoresistance.

In conclusion, our study indicates that PAK6 decreases 5-FU drug susceptibility in colon cancer cells and is an independent prognostic factor for adjuvant 5-FU-based chemotherapy in patients with stage II and stage III colon cancer. This suggests that at the time of an initial colon cancer diagnosis, PAK6 expression might be used to not only identify the optimal individualized treatment, but also to distinguish patients who are more likely to benefit from chemotherapy after surgery.

## Methods

### Patients and clinical database

This study used tumor specimens from 147 stage II and III colon cancer patients, enrolled from January 2001 to December 2003, who underwent radical resection and received 5-FU-based chemotherapy after surgery. The clinicopathological factors of all the patients are presented in Table 1. The patients were followed by history and physical surveillance every 3 months for 2 years, then every 6 months up to 5 years, and finally annually. Written informed consent was obtained from each patient before entering the study and all study protocols were approved by the Ethics Committee for Clinical Research of Shanghai Jiaotong University affiliated Shanghai First People's Hospital Medical Center.

### IHC

Immunostaining was performed using a GT Vision III Kit (Genetech, Shanghai, China). After antigen retrieval in citrate buffer (pH 6.0) for 10 min, specimen slides were incubated overnight at 4°C with the primary antibody against PAK6 (1:100, Cell Signaling Technology, USA). The slides were then incubated with the secondary antibody (Genetech, Shanghai, China) for 30 min at room temperature. Tissue sections were counterstained with Mayer's hematoxylin. The intensity and extent of staining was independently evaluated by 2 pathologists who were blinded to patient outcomes. The staining intensity was scored as 0 (no staining), 1 (mild staining), 2 (moderate staining), or 3 (intense staining). The staining area was scored as 0 (0%), 1 (1%–25%), 2 (26%–50%), 3 (51%–75%), or 4 (76%–100%) according to the percentage of positively-stained cells. The final staining scores, calculated by the sum of the intensity and area scores, were divided into 3 groups as follows: 0–2, negative expression; 3–4, weak expression; and 5–6, strong expression. In cases where there was a discrepancy between the pathologist's assessments, the slides were re-examined by both pathologists under a multi-head microscope until an agreement was reached.

### Cell lines and reagents

The human colon carcinoma cell lines HCT8, HCT116 and SW480 were purchased from the American Type Culture Collection. Cells were maintained in Dulbecco's modified Eagle's medium supplemented with 10% fetal bovine serum (Gibco) and cultured at 37°C with 5% CO_2_. 5-FU was purchased from Sigma (St Louis, MO, USA).

### Establishment of PAK6-KD cell lines

Commercially available PAK6 shRNA constructs were obtained (Genechem Co. Ltd., Shanghai, China) and used to silence PAK6. The PAK6 shRNA duplex sequences are as follows:

KD1 forward: TAGTGATCTCCAGGTCTTTGTATCGAGTACAAAGA CCTGGAGATCACTTTTTTTC,

KD1 reverse: TCGAGAAAAAAAGTGATCTCCAGGTCTTTGTACT CGAGTACAAAGACCTGGAGATCACTA,

KD2 forward: TAGATCAGCAAAGACGTCCCTACTCGAGTAGGGA CGTCTTTGCTGATCTTTTTTTC,

KD2 reverse: TCGAGAAAAAAAGATCAGCAAAGACGTCCCTAC TCGAGTAGGGACGTCTTTGCTGATCTA.

HCT8 and HCT116 cells were transduced with 5 × 10^5^ transducing units/mL of lentivirus particles. The transduction efficiency was 87% and 92% for HCT8 and HCT116 cells, respectively. Antibiotic selection (1 μg/mL puromycin) was initiated for 7 days 24 h after transduction. As a result, 2 HCT8 PAK6-KD stable cell lines (HCT8 PAK6-KD1 and PAK6-KD2) and 2 HCT116 PAK6-KD stable cell lines (HCT116 PAK6-KD1 and PAK6-KD2) were generated. Cells transduced with shNT (HCT8 shNT and HCT116 shNT) were used as controls.

### Establishment of PAK6 overexpressing cell lines

To restore the expression of PAK6 in SW480 cells, human full length *PAK6* cDNA was inserted into the GV230 vector (Genechem Co. Ltd., Shanghai, China), which was transduced into SW480 cells using Lipofectamine 2000 (Invitrogen) in accordance with the manufacturer's advised procedure. The empty vector was transduced into control cells. The PAK6 expressing and control cells were designated SW480/PAK6 and SW480/vector, respectively.

### RNA extraction and qPCR

qPCR analysis was performed on all of the cell lines and on the 40 frozen tumor tissue and corresponding normal tissue specimens. Total RNA was extracted and qPCR was performed as previously described [35]. qPCR analysis of *PAK6* expression was conducted using 2 μL of cDNA. The forward primer sequence was 5′ atctgggagagaaggcgaac 3′ and the antisense primer sequence was 5′ ggaggacaaggagacagcag 3′. β-actin was used as an internal control. All of the experiments were performed at least in triplicates.

### Protein extraction and western blotting

Western blotting was performed as previously described [35]. Briefly, membranes were incubated with primary antibodies targeting PAK6, cleaved PARP, cleaved caspase 3, serine112 p-BAD, total BAD, or β-actin overnight (1:1000, Cell Signaling Technology, USA). Following overnight incubation with the primary antibody, membranes were washed 3 times in tris buffered saline-Tween 20 (TBST) for 10 min each. The membranes were then incubated with the secondary antibody in 5% milk in TBST for 1 h. Following the secondary antibody incubation, membranes were washed for 30 min, and the targeted proteins were detected by chemiluminescence (Pierce Biotechnology, Rockford, IL, USA). β-Actin levels were used as loading controls.

### Cell viability assay

To analyze the viability of cells treated with 5-FU and with altered PAK6 expression, 1 × 10^4^ cells/well were seeded in 96-well plates containing 0.2 mL of medium. After treatment, MTT (5 mg/mL, Sigma, USA) was added to each well (including the control well) and the mixture was incubated at 37°C for 2 h. The culture medium was then replaced with an equal volume of dimethyl sulfoxide (Sigma, USA). After shaking at room temperature for 10 min, the 490 nm absorbance (A490) of each well was determined on a microplate reader (Bio-Rad, USA). Cell viability was calculated according to the following formula: cell viability (%) = A490 (sample)/A490 (control) × 100.

### Flow cytometry analysis of the cell cycle and apoptosis

The cell cycle was analyzed using the Cell Cycle Kit (BD, CA, USA). Cells were trypsinized and washed in phosphate buffered saline (PBS) for 5 min prior to collection by centrifugation at 1500 revolutions per minute (rpm). Cells were then fixed with 2 mL of prechilled 70% ethanol for 30 min at 4°C. The cells were then gently centrifuged and resuspended in a solution containing RNase A and PI. After 30 min incubations, the cells were analyzed by flow cytometry (BD Accuri C6, USA). For the apoptosis assay, the cells were trypsinized and washed with serum-containing medium. Cells were then centrifuged for 3 min at 1500 rpm and the supernatant was discarded. The cells were resuspended in 1 × Binding Buffer at 1–5 × 10^6^/mL. The cells were then stained for 10–15 min at room temperature using the Annexin V-APC/PI Apoptosis Kit (eBioscience, CA, USA) in accordance with the manufacturer's instructions. The number of apoptotic cells was analyzed using flow cytometry (BD Accuri C6, USA).

### Nude mice xenograft models

Colon cancer xenografts were established in 4-week-old male BALB/C nude mice purchased from the Institute of Zoology, Chinese Academy of Sciences of Shanghai. Briefly, PAK6-KD and shNT cells (3 × 10^6^) were suspended in 100 μL of PBS and injected subcutaneously into the flank of the 4-week-old nude mice. The tumor volume was calculated using the formula: length × width^2^ × 0.5. When the tumor volumes were approximately 0.2 cm^3^, the mice were divided into 4 groups: shNT, shNT plus 5-FU, shKD, and shKD plus 5-FU. 5-FU was dissolved in PBS and 20 mg/kg/day was injected intraperitoneal twice a week for 3 weeks. The tumor volume and body weight of the mice were measured once a week and the mice were sacrificed after 5 weeks. All of the animal procedures were conducted in accordance with the Shanghai Jiaotong University Affiliated Shanghai First People's Hospital Animal Care guidelines. All efforts were made to minimize animal suffering.

### Statistical analysis

The significance of the *in vitro* and *in vivo* data was determined using the Student t test (2-tailed). The 2-tailed χ^2^ test and Fisher exact test were used to determine the statistical significance of the covariate differences. Survival rates were calculated using the Kaplan–Meier method and the log-rank test was used to compare the survival curves. The Cox proportional hazards model was used to calculate multivariate HRs for the variables. A *P* value of less than 0.05 was considered to be statistically significant. All statistical analyses were carried out using the SPSS 19.0 statistical software package (SPSS Inc., Chicago, IL).

## SUPPLEMENTARY FIGURES AND TABLE


