# Supplementary material for: PAK6 increase chemoresistance and is a prognostic marker for stage II and III colon cancer patients undergoing 5-FU based chemotherapy
Source: Oncotarget. 2014 Nov 7;6(1):355–67. doi: 10.18632/oncotarget.2803 (PMC4381600; doi:10.18632/oncotarget.2803)
Supplement: Supplementary file 1 [file oncotarget-06-355-s001.pdf]

PAK6 increase chemoresistance and is a prognostic marker for stage II and III colon cancer patients undergoing 5-FU based chemotherapy

Supplementary Material

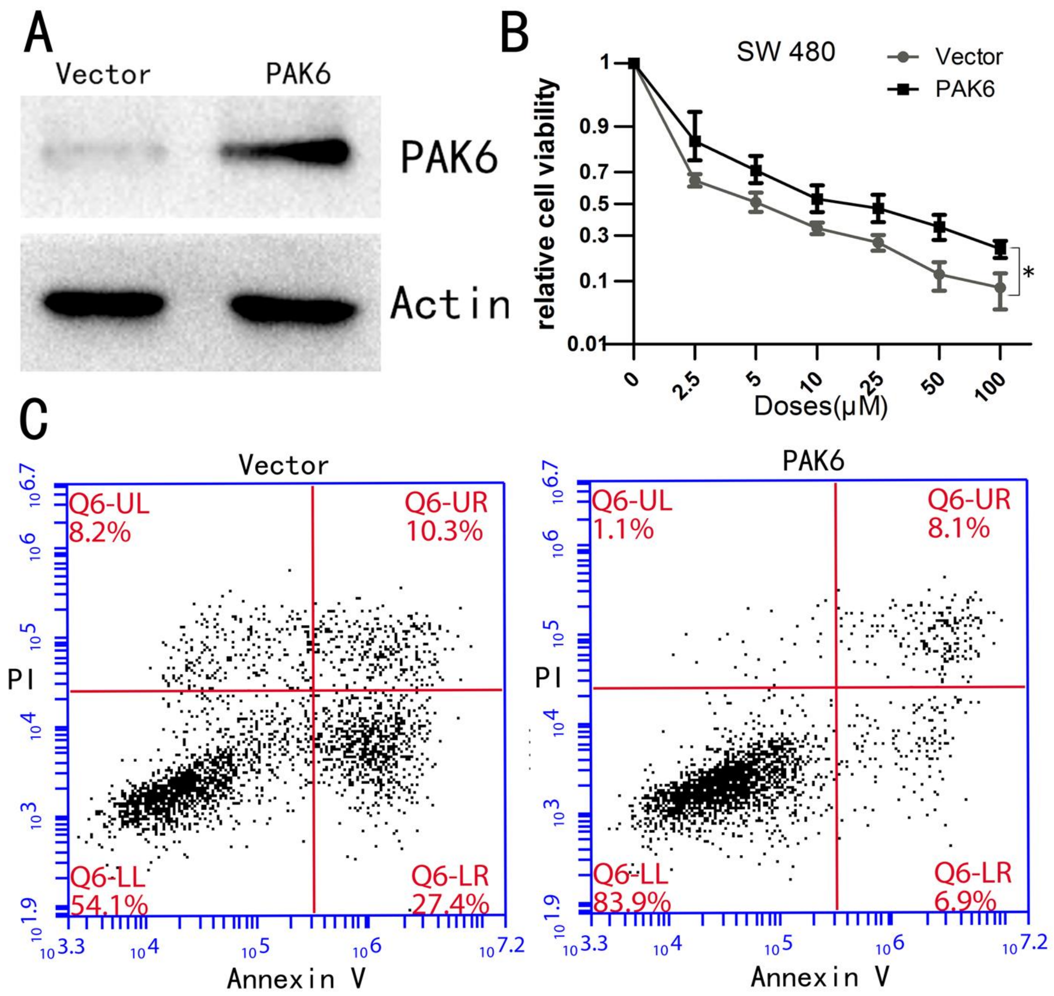

Supplyment figure 1: Cell viability and cell apoptosis in PAK6 overexpressed SW480 cells. A: SW480 cells were overexpressed with PAK6. B: SW480 were treated with 5-FU for 48h, SW480/ vector IC50 = 5.19 ± 0.02uM, SW480/ PAK6 IC50 = 18.23 ± 0.04uM (* $P < 0.05$). C: the apoptosis rate of SW480/ PAK6 is significantly less than the SW480/ vector control (SW480/ vector=27.38±0.86, SW480/ PAK6=7.69±0.14, $P < 0.05$).

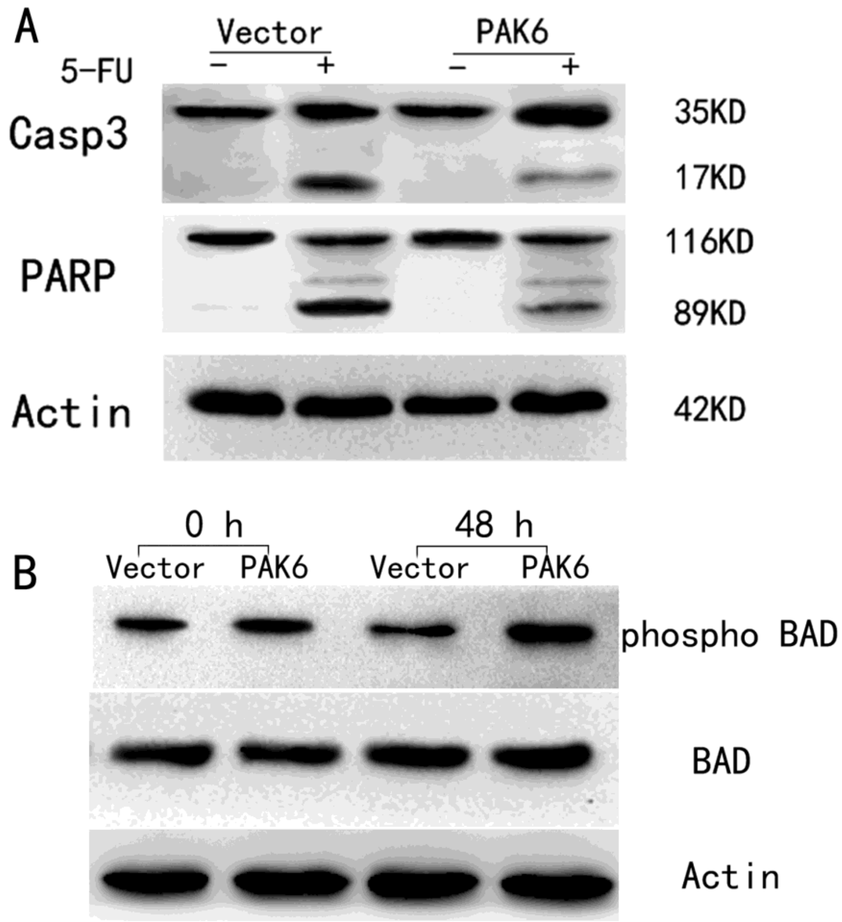

Supplyment figure 2: Changes of apoptosis-related molecules after a 48-h treatment with 10 uM 5-FU in SW480. A: Western blot analysis showed that caspase 3 and PARP were less cleaved in PAK6-overexpressed SW480 cell lines. B: Phosphorylated BAD (p-BAD) was increased in PAK6-overexpressed SW480 cells.

Supplyment table 1: Percentage of cells in each phase presented.

treatment	shNT		ShNT+5-FU		shKD		ShKD+5-FU	
Cell type	HCT8	HCT116	HCT8	HCT116	HCT8	HCT116	HCT8	HCT116
Sub-G0/G1	**0**	**0**	**0.20±0.03**	**0.53±0.07***	**0**	**0.37±0.09***	**6.40±0.06***	**7.57±0.37***
G0/G1	**72.97±0.20**	**76.27±0.52**	**58.40±0.23***	**59.40±0.1***	**61.1±0.15***	**60.13±0.03***	**32.57±0.15***	**34.4±0.51***
S	**16.07±0.20**	**12.2±0.27**	**25.60±0.17***	**24.00±0.27***	**14.00±0.12**	**18.13±0.20**	**18.63±0.13**	**18.93±0.20**
G2/M	**10.97±0.03**	**11.43±0.29**	**15.67±0.03**	**16.07±0.13**	**24.90±0.06***	**21.37±0.09***	**42.40±0.12***	**39.1±0.06***

*P < 0.05 indicates a significant difference compared with shNT group.
